# First Documented Fatal Gastric Obstruction Associated with Ingestion of Plastics and Vegetation in a Juvenile *Kogia breviceps*

**DOI:** 10.3390/ani16142194

**Published:** 2026-07-15

**Authors:** Denis Benito, Andrea Estarrona, Maider Iturrondobeitia, Julen Ibarretxe, Nahiara Muguerza, Irune Valenciano, Xabier Lekube, Manu Soto, Urtzi Izagirre

**Affiliations:** 1 Research Centre for Experimental Marine Biology and Biotechnology (PiE-EHU), University of the Basque Country (EHU), 48940 Plentzia, Basque Country, Spain; andrea.estarrona@ehu.eus (A.E.); maider.iturrondobeitia@ehu.eus (M.I.); julen.ibarretxe@ehu.eus (J.I.); nahiara.muguerza@ehu.eus (N.M.); irune.valenciano@ehu.eus (I.V.); xabier.lecube@ehu.eus (X.L.); manu.soto@ehu.eus (M.S.); urtzi.izagirre@ehu.eus (U.I.); 2 CBET+ Research Group, Department of Zoology and Animal Cell Biology, Faculty Science and Technology, University of the Basque Country (EHU), 48013 Bilbao, Basque Country, Spain; 3 Life Cycle Thinking Group, Department of Graphic Design and Engineering Projects, Faculty of Engineering in Bilbao, University of the Basque Country (EHU), 48013 Bilbao, Basque Country, Spain; 4 Life Cycle Thinking Group, Department of Applied Physics, Faculty of Engineering in Bilbao, University of the Basque Country (EHU), 48013 Bilbao, Basque Country, Spain; 5 Laboratory of Botany, Department of Plant Biology and Ecology, Faculty Science and Technology, University of the Basque Country (EHU), 48013 Bilbao, Basque Country, Spain

**Keywords:** pygmy sperm whale, marine litter, gastric obstruction, starvation, stranding

## Abstract

The stranding network of the Basque Country (SAREUS) attended a stranded juvenile female *Kogia breviceps* on Zarautz (Gipuzkoa, Spain) on 2 October 2025. The necropsy revealed severe pathological findings: (1) plastic and plant debris occupying most of the volume of the main stomach, (2) pulmonary congestion, pink froth in respiratory airways and presence of sand in the oesophagus, and (3) haemorrhagic liver and meninges together with serosanguineous liquid in the pericardium. Results indicate that the individual stranded alive and drowned because of a debilitated condition most likely caused by plastic and plant material ingestion. To understand what happened in the last days of the animal, and to identify behaviours that could lead to plastic ingestion, the stomach content was analysed, including the chemical characterization of the plastic debris through ATR-FTIR and the identification of vegetal and prey species. One possible explanation of the stranding event could have been that reduced foraging ability in a juvenile animal contributed to ingestion of foreign material, although this hypothesis cannot be confirmed from the available evidence.

## Simple Summary

This case report addresses the impact of marine pollution on marine mammals by examining the case of a juvenile pygmy sperm whale found stranded on the coast of the Bay of Biscay in northern Spain. The main aim was to determine the cause of death through a detailed examination of the animal and the analysis of its stomach contents. The results showed the presence of a large amount of plastic debris and plant material blocking the stomach, which prevented proper feeding and led to severe physical deterioration. Evidence also indicated that the animal stranded alive and subsequently drowned. The determination of the cause of death and the bibliography on the subject support the hypothesis that the juvenile could have ingested great quantities of non-food material due to its limited ability to identify suitable prey, possibly due to its early age and lack of foraging experience. The importance of these findings show how marine litter, particularly plastic waste, can have direct and fatal consequences for marine mammal species; furthermore. This is the first report of a pygmy sperm whale ingesting plant material. Overall, this study contributes to a better understanding of the environmental threats unknown species such as Kogiids face and gives critical information on stranding causes.

## 1. Introduction

The accumulation of marine macroplastics has emerged as a critical driver of ecosystem degradation, as large synthetic debris increasingly permeate every oceanic compartment from the surface to the deep sea [1]. Beyond its presence as an inert pollutant, macro-scale litter acts as a significant physical stressor that disrupts the biological functions of marine organisms through ingestion and entanglement, affecting over 700 species worldwide [2]. Recent studies highlight that these materials not only compromise the survival of marine megafauna but also serve as artificial substrates for the long-distance dispersal of invasive species and pathogens, potentially altering the genetic and structural integrity of native marine communities [3]. Consequently, the persistence of these non-biodegradable polymers represents a systemic threat to the delivery of essential ecosystem services and the long-term resilience of the global ocean [4].

Gastrointestinal obstruction is a primary fatal outcome of foreign body ingestion in cetaceans, frequently involving materials such as plastic debris [5,6]. Beyond direct mortality, these contaminants induce sublethal effects by compromising foraging and digestive capabilities, disrupting hunger signalling, and hindering predator avoidance. Furthermore, such ingestion can inhibit reproduction and growth, reduce body condition, and impair locomotion, including migratory behaviour [7,8]. Plastic debris ingestion has been documented across 58% of all cetacean species—affecting both odontocetes and mysticetes—with a prevalence of up to 31% in certain populations [9,10]. Among these, deep-diving species are suggested to be particularly vulnerable, likely due to their specialised foraging strategies at great depths [11,12]. Indeed, the seafloor serves as a major sink for marine litter, exhibiting a high concentration of plastic items [8,13,14].

A less documented cause of death in cetaceans involving foreign body ingestion is digestive tract impaction by plant material. While several studies report the presence of botanical material in the stomachs of odontocetes, including harbour porpoises [15], northern right whale dolphins [16], spinner dolphins [17], and bottlenose dolphins [5], only a few have identified this phenomenon as the cause of gastric impaction leading to pyloric failure and subsequent death [5,17,18]. Various hypotheses explain the ingestion of both plastics and plant material; one of them suggests that juvenile individuals suffering from maternal absence may exhibit reduced predatory capacity and low echolocation efficiency, leading to the ingestion of these foreign bodies as a compensatory mechanism for satiety when unable to get adequate prey [5,11]. In fact, previous studies [12] have described significant higher prevalence of foreign body ingestion in juvenile stranded cetaceans when compared to adults.

Deep-diving habits make the genus Kogia (Family Kogiidae) particularly vulnerable to plastic ingestion, with records highlighting them as one of the affected marine mammal groups. This genus comprises two small odontocete species: the pygmy sperm whale (*Kogia breviceps*) and the dwarf sperm whale (*Kogia sima*). These elusive cetaceans inhabit warm-temperate to tropical oceans, particularly along continental slope regions. Given that they are rarely observed alive, most biological knowledge is derived from stranding data and necropsy findings [19]. *K. breviceps* can reach lengths of 4.3 m and weights of up to 515 kg [20]. Their diet consists primarily of cephalopods, supplemented by fish and crustaceans [21]. Studies worldwide have reported the negative impacts of plastic ingestion on *K. breviceps* [11,16,21,22,23,24,25,26,27]. Although the harm that plastic debris ingestion can cause in *K. breviceps* has been previously described [11,24], there are few reported cases in which a stomach obstruction has been described as the main cause of death [11,23]. In the cited works, the most remarkable pathologies associated with this phenomenon were ulcerative gastritis and gastric obstruction that could result in impaired digestion, malnutrition, and increased susceptibility to comorbidities, among others. However, along the northern coast of the Iberian Peninsula, records of plastic ingestion in stranded *K. breviceps* individuals have only suggested sublethal consequences and minor effects [8].

Thus, the aim of this work is to describe the stranding event of an uncommon species that was caused by a combination of factors that has not been reported before, as far as the authors are aware, in the northern Iberian Coast. For that purpose, the interpretation of the gross pathologies discovered in the necropsy and the thorough analysis of the stomach content, including taxonomic identification of biological material, as well as the chemical characterisation of the plastic debris found obstructing the digestive track were performed. It is necessary to consider that although necropsies have been proven to be useful to give insights into the morbidity of an individual and the historical pressures the animal has faced over its lifetime [28], some of the interpretations drawn from the forensic results, mostly the behavioural ones, cannot be confirmed and can be conjectural. Thus, to address the knowledge gaps regarding the elusive pygmy sperm whale, continued research and systematic reporting of these stranding events and subsequent post-mortem analyses remain essential.

## 2. Materials and Methods

### 2.1. Stranding Event, Carcass Conservation and Necropsy Procedure

**Figure 1:**
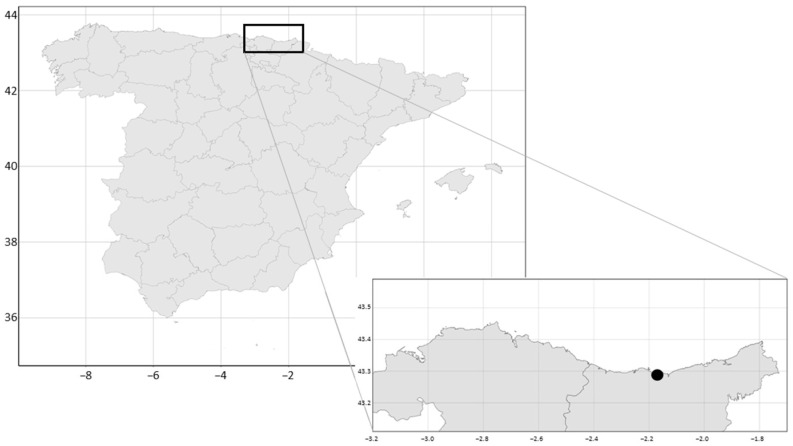
Map of the location of the stranding event in Zarautz, Gipuzkoa. Border lines indicate provinces of the Spanish state.

**Figure 2:**
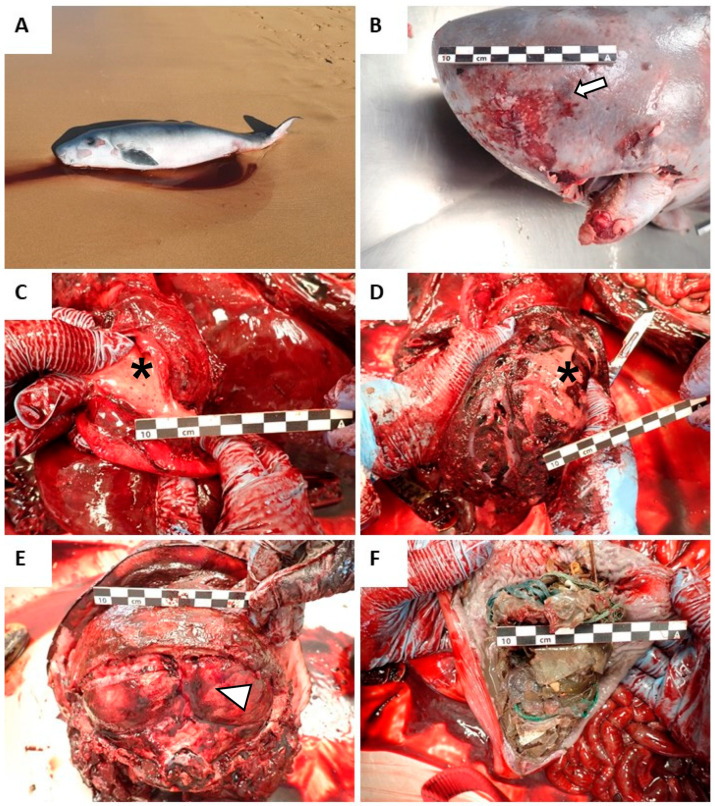
Photographs of the stranded *K. breviceps* and main pathological findings. (**A**) *K. breviceps* as found when the stranding network crew arrived to the beach; (**B**) rostral area presenting the most remarkable external lesions; (**C**) trachea full of pink froth; (**D**) bronchi expulsing pink froth when pressure was applied on lung parenchyma; (**E**) haemorrhagic meninges and (**F**) severely impacted keratinized stomach full of non-food items. Scale bars: Each white or black rectangle measures 1 cm in length. Arrow: rostral lesions; asterisks: pink froth in airways; arrowhead: haemorrhages.

On 2 October 2025 on Zarautz beach (43°17′17.9″ N 2°10′06.2″ W), Gipuzkoa, Spain (Figure 1), a juvenile female *Kogia breviceps* specimen measuring 160 cm and weighing 51.1 kg (Figure 2A) stranded dead. The carcass was transported to the Plentzia Marine Station (PiE-EHU) by SAREUS staff, where it was stored at 4 °C for four days prior to necropsy. The necropsy was performed following the protocol from the Spanish Ministry of Ecological Transition and Demographic Challenge [29] (images of the main three stages of the necropsy are available as Supplementary Figure S1A–C) and was carried out by the scientific staff of the stranding network of the Basque Country (SAREUS) and a Tragsatec veterinarian hired as part of a commission from the Ministry of Ecological Transition and Demographic Challenge (MITECO). The necropsy report included biological data, species identification, biometrics, age, sex, body condition and conservation status [30]. In the internal analysis, the inspection of the different systems located in the thoracic and abdominal cavities was done together with the determination of the presence of ecto- and endoparasites. Within the thoracic cavity, the circulatory and respiratory systems were examined, including the heart, major blood vessels, trachea, lymph nodes, and lungs. In the abdominal cavity, the following systems were analysed: the digestive system (mouth, oesophagus, stomach, intestines, pancreas, adrenal glands, liver and spleen), the reproductive system (ovaries), and the excretory system (kidneys and bladder). Representative samples of these tissues and organs were collected and frozen at −80 °C for future toxicological analysis and molecular identification of viral and bacterial pathogens. Samples for histological processing and subsequent histopathological analysis were also retrieved. Additionally, other organs such as the inner ears and the encephalon were also examined.

### 2.2. Stomach Content Analysis

The stomach content of the individual was collected for the analysis of different items. The entirety of the keratinized stomach content was transferred to a stainless steel tray, and the objects were carefully extracted and grossly classified in three categories: food items, plant material and plastics. The food items were separated depending on the degradation level of the remains, and afterwards all the biological material was stored in ethanol 70° at 4 °C. The most degraded food material and the plant material were analysed under the stereoscope to confirm the animal/botanical origin. The abundance of crustaceans was estimated using the carapace and claw numbers. As a reference for the taxonomic identification of the crustaceans, the work of Zariquiey-Álvarez (1968) [31] was used. The presumptive non-food items were separated as plant material and plastic items. For the taxonomic determination of the plant material, well-preserved macroscopic remains were identified de visu based on diagnostic morphological characteristics, following Ruiz et al. (2015) [32]. The weighing of the biological material was performed after extracting the samples from the ethanol and eliminating the excess of liquid without permitting complete desiccation to avoid damage to potential taxonomic characters. Each of the plastic items found in the stomach of the individual was carefully cleaned in the surface and weighed; additionally, a probable origin or use of the plastics was determined according to morphological characteristics, or even written text and drawings present on them. The plastics were then analysed by attenuated total reflectance Fourier-transformed infrared (ATR-FTIR) spectroscopy. Infrared spectra were acquired directly using an Agilent 4300 spectrometer (Santa Clara, CA, USA) operating in attenuated total reflectance mode. After calibrating the equipment before each measurement, the infrared spectra were acquired in the 650–4000 cm^−1^ range with a spectral resolution of 4 cm^−1^, accumulating 64 scans to achieve a good signal-to-noise ratio. In general terms, the acceptance threshold or high quality index (HQI) was 80%, except in the cases where sea water degradation and absorption occurred, where a value over 70% was accepted (Supplementary Table S1). Although some degree of weathering was present, the main issue was the disintegration due to the effect of seawater, which was visible at ATR spectra, where the band related to O-H bonds and salt presence increased as the HQI decreased (Supplementary Figure S2A–C). Two spectra were collected per plastic fragment and then one of them was selected, each presenting similar measurements in all cases. The database used to determine the polymer grade was KnowItAll IR Spectral Library included in Wiley’s KnowItAll Spectral Software (v2026.1, John Wiley & Sons, Inc., Hoboken, NJ, USA).

## 3. Results

### 3.1. Necropsy Report

The decomposition state of the carcass was determined to be moderately decomposed (code 3). The external lesions found were mainly located in the frontal area, mostly concentrated in the rostral area (Figure 2B) and in the ventral side of the animal. No ecto- or endoparasites were detected. The carcass presented a poor nutritional status, and the thickness of blubber was measured from the lateral zone of the body, in a vertical transect between the pectoral and dorsal fins: 23.4 mm in the dorsal section, 12.4 mm in the ventral section and 17.1 mm in the lateral section. The most remarkable lesions regarding the examination of the internal organs revealed the presence of serohaemorrhagic fluid in the pericardium, pink froth in the trachea (Figure 2C) and bronchi (Figure 2D), congestive lungs, and haemorrhagic liver and meninges (Figure 2E). Interestingly, sand was discovered in the oesophagus, and the keratinized stomach presented a severe impaction caused by plastic debris and plant material (without associated lacerations) together with prey remains (Figure 2F). These objects caused a complete obstruction of the digestive tract as the glandular and pyloric stomachs were empty, and only a small amount of faecal material was found before the “ink sac”. The necropsy report concluded that the most probable cause of death was the gastric obstruction.

### 3.2. Stomach Content

The total weight of the material retrieved from the stomach was 126.61 g, divided by 50.31 g (39.74%) of prey remains, 31.10 g (24.56%) of plant material and 45.20 g (35.70%) of plastic items, as summarised in Table 1.

**Figure 3:**
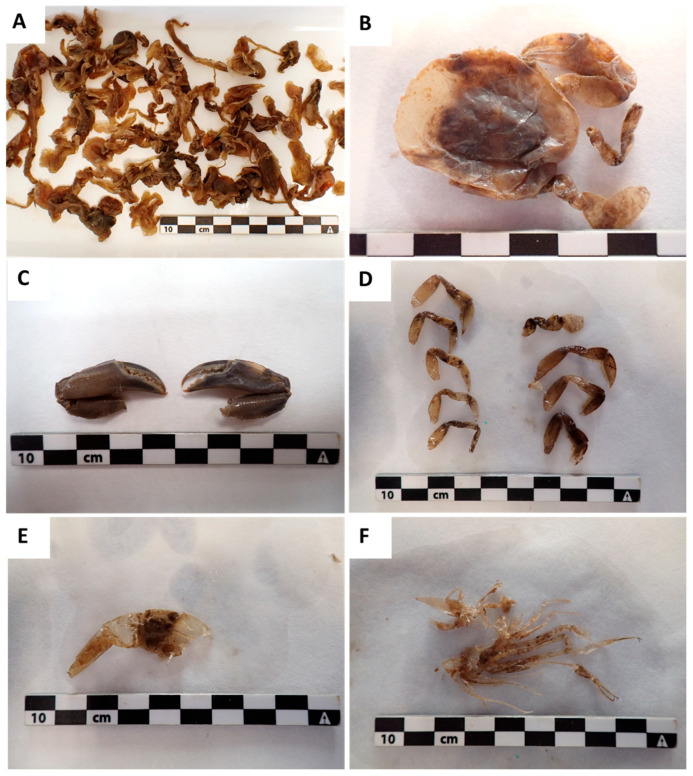
Photographs of food remains from the stomach of the stranded *K. breviceps*. (**A**) Mass of unidentifiable crustacean cuticles; (**B**) cephalotorax, claw, walking leg and swimming leg belonging to *P. henslowii*; (**C**) pair of claws belonging to *P. henslowii*; (**D**) walking and swimming legs from different *P. henslowii* individuals; (**E**) cuticle remains from *P. multidentata* abdomen; (**F**) cuticle remains from the first appendixes of a *P. multidentata*. Scale bars: Each white or black rectangle measures 1 cm in length.

The only food items retrieved from the stomach of the stranded *K. breviceps* were the remains of crustaceans. The largest fraction was a mass of unidentifiable cuticle remains (Figure 3A) weighing 45.34 g. Additionally, identifiable body parts of at least three *Polybius henslowii* crabs (Figure 3B–D) and one *Pasiphaea multidentata* shrimp (Figure 3E,F), weighing 4.03 g and 0.94 g, respectively, were retrieved from the stomach.

**Figure 4:**
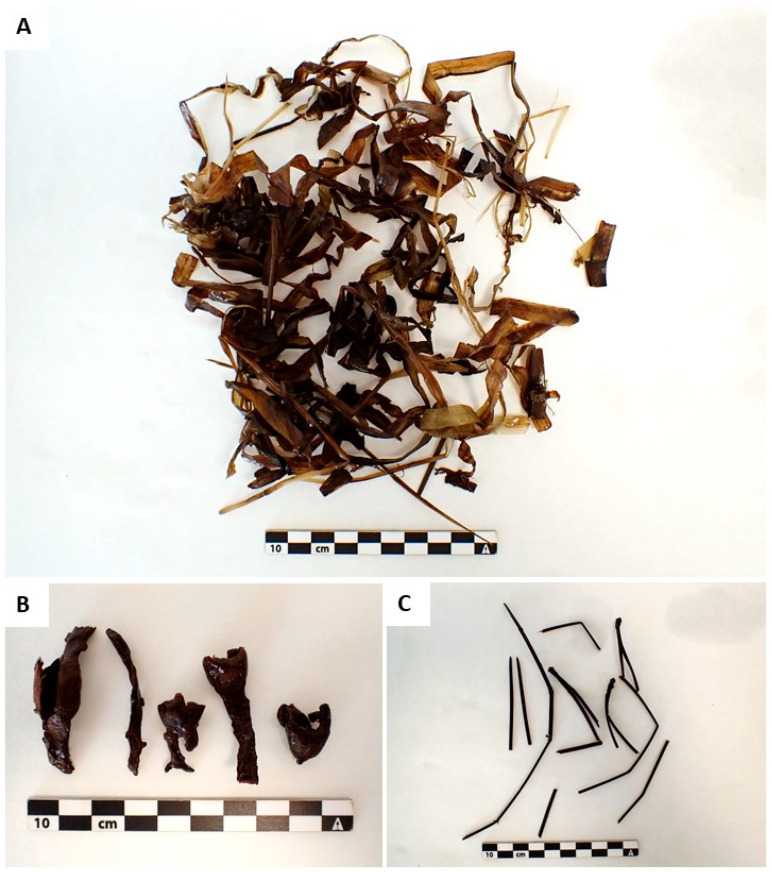
Plant material recovered from the keratinized stomach of the pygmy sperm whale stranded in Basque Coast. (**A**) *Zostera marina*; (**B**) *Ascophyllum nodosum*; and (**C**) unidentified plant fragments. Scale bars: Each white or black rectangle measures 1 cm in length.

The stomach content of the pygmy sperm whale presented three distinct types of plant material (Figure 4): (A) *Zostera marina* (23.02 g), (B) *Ascophyllum nodosum* (4.23 g), and (C) other highly degraded plant fragments (3.85 g) that could not be reliably identified (for more detailed images: Supplementary Figure S3A–C) possibly including rushes or other coastal vegetation.

**Figure 5:**
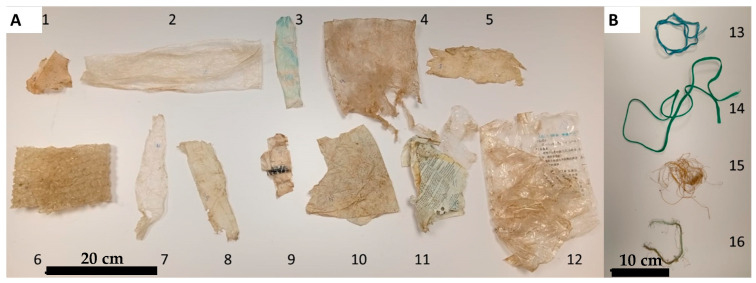
Plastic fragments analysed by ATR-FTIR. Scale bars: (**A**) 20 cm approx., (**B**) 10 cm approx. Numbers correspond with the sample number in Table 2.

ATR-FTIR analysis was performed on 16 plastic fragments retrieved from the stomach of the stranded pygmy sperm whale (Figure 5), 15 of them being characterised as low-density polyethylene (LDPE) and 1 as part of the polyethylene family in general, containing acrylic traces; detailed information is presented in Table 2. A spectrum example and the data extraction procedure are summarised in Supplementary Figure S4, where the measured wave numbers and corresponding intensities were as follows:

2915 at 0.462; · 2848 at 0.373; · 1465 at 0.159; · 1377 at 0.039; · 719 0.209; · 730 0.098

Main bands matching with KnowItAll database indicated the following:

- 2911.1 cm^−1^*—*Aliphatic C–H stretching (–CH_2_–)
- 2844.0 cm^−1^*—*Aliphatic C–H stretching
- 1464.8 cm^−1^*—*CH_2_ bending (scissoring)
- 713.8 cm^−1^*—*CH_2_ rocking (–(CH_2_)_n_–)
- 1371.7 cm^−1^*—*CH_3_ bending (branching)

All together, the results suggested that the plastic could be classified as low-density polyethylene.

## 4. Discussion

The present study gives novel information on a relatively unknown species such as *Kogia breviceps*, as it is the first reported case of stomach impaction caused by plant and plastic debris as the main cause of stranding and death in this species. The evidence that pollutants of anthropogenic origin were partially the cause of the gastric obstruction that led to the extreme weakening and death of the studied animal underscores the necessity to continue researching and acting against a well-known global problem such as marine plastic pollution.

The Kogiidae family is still one of the least studied odontocete taxa due to their elusive and deep-diving behaviour; consequently, the population trends of *K. breviceps* remain unknown, and the majority of the information known to date has been obtained from stranded individuals [11]. Similarly, the presence of these two species in the Basque Coast has been scarce, the stranding event described herein being the sixth stranded *K. breviceps* individual since 1998. Unfortunately, until the stranding network of the Basque Country started to be managed by SAREUS and PiE-EHU in the summer of 2022, no systematic necropsy and sample collection protocols were implemented in the region, making it impossible to research the previous *K. breviceps* stranding events. It is worth mentioning that in 2025, after the stranding event from October studied herein, another *K. breviceps* individual stranded at the start of December and the first ever *K. sima* in the Basque Coast stranded less than three weeks afterwards. Although having three Kogiid stranding events in a time span of less than 3 months in such a small region (246 km of coastline) could seem alarming, the aetiology of the three events seems to be unconnected. Nevertheless, further research needs to be performed to rule the cause of death of the last two individuals.

The pathological characteristics described in the necropsy indicate that the starvation caused by the stomach impaction made the animal suffer a pathological consumptive process that ended when the animal drowned on the shoreline. The external lesions described herein are concordant with the stranding event, being caused by the collision against the shore [6], and do not correspond with the characteristic signals found in other anthropogenic interactions like vessel strikes or bycatch [33]. In addition to the poor body condition observed, the thickness of the blubber suggests an ongoing emaciation process. Previous studies [34] reported a mean blubber thickness of 27.98 ± 1.35 mm in seven adult *K. breviceps* individuals with good body condition, which is far superior to the 17.1 mm reported here in the same location (lateral section). The generalised haemorrhagic and congestive condition described in the necropsy are coherent with the stress generated by a live-stranding event [6], and the presence of sand in the oesophagus and pink froth in the airways would indicate that the pygmy sperm whale arrived alive to the shore and drowned afterwards [35]. The characteristics of the present case coincide with a report of a live-stranded Risso’s dolphin that died on shore, in which stomach obstruction by plastics was determined to be the cause triggering the stranding event [36,37].

In addition to the elucidation of the cause of death related to the gastric obstruction of the studied individual, the analysis of the stomach content enabled obtaining information about the diet of the species and confirmed the presence of plant debris, the latter being the first report in this species as far as the authors are aware. As will be discussed below, this proved to be symptomatic of altered feeding behaviour.

It has been described that the preferred prey of Kogiids are cephalopods, followed by fish, and lastly crustaceans [21,38]. However, the food items found in the present study are not what would be expected. The two crustacean species identified in the present study have been described before as part of the diet of *K. breviceps*: (1) Santos et al. (2006) [39] reported the presence of 29 *P. henslowii* crabs in a pygmy sperm whale stranded in France and concluded that the neritic characteristics of this decapod could be indicative of abnormal hunting behaviour associated with the stranding of the animal. (2) West et al. (2009) [38] reported the presence of shrimps from the genus *Pasiphaea* in the stomach of *K. breviceps* individuals in low mass percentages, although the deep-water ecology of *P. multidentata* [40] makes these shrimps compatible as complementary prey for the deep-diving ecology of Kogiids. Thus, the lack of most common prey could indicate a predatory incapacity in the studied individual.

The wide distribution of the prey species retrieved from the necropsied individual does not give much evidence regarding the whereabouts of the stranded individual before its death; however, the plant debris found in the stomach could give some additional information. The species that was retrieved in largest quantity was *Zostera marina*, a marine angiosperm that develops on sandy–muddy substrates in shallow, sheltered coastal environments, where high light availability, low hydrodynamic energy, and relatively stable sediments allow the formation of persistent seagrass meadows [41,42]. Along the Cantabrian coast, the distribution of *Z. marina* is scarce and highly fragmented. Although the species has been reported from Galicia to Cantabria, its occurrence is irregular and populations are generally small and spatially isolated [43]. *Z. marina* is also widely distributed along the French Atlantic coast, particularly in Brittany and in sheltered estuarine systems of southwestern France, where extensive intertidal and shallow subtidal meadows develop on soft sediments [44]. In contrast, along the open Basque coast, environmental conditions characterised by high wave exposure and limited sheltered embayments prevent the establishment of self-sustaining populations. Consequently, records from this region are largely restricted to detached leaves or floating material transported by coastal currents and storm-driven processes [45].

The second vegetal species identified was *Ascophyllum nodosum*, a long-lived North Atlantic intertidal brown alga that typically develops on sheltered to moderately exposed rocky shores within the mid-intertidal zone. Its natural distribution extends from northern Norway and the British Isles to northern Portugal, including the Galician and Asturian sectors of the Cantabrian Sea, where it forms localised but stable populations under suitable hydrodynamic conditions [32,46]. Along the French Atlantic coast, the species is widespread and abundant, forming dense and persistent intertidal belts that structure coastal ecosystems and support high associated biodiversity [47]. Conversely, along the open Basque coast, *A. nodosum* does not form stable populations. Observations are limited to detached fronds and floating thalli transported from western source populations [48]. Taken together, the restricted local occurrence of both taxa, their well-established populations to the west and northeast of the study area, and the dominant west-to-east surface circulation in the southeastern Bay of Biscay strongly support an interpretation of allochthonous origin for the plant material recovered from the stomach of the studied pygmy sperm whale.

The ingestion of marine debris by *K. breviceps* has been documented in several regions worldwide [11,16,21,22,23,24,25,26,27]. Although the harm that plastic debris ingestion can cause in *K. breviceps* has been previously described [11,24], there are few reported cases in which a stomach obstruction has been described as the main cause of death [11,23]. In the cited works, the most remarkable pathologies associated with this phenomenon were ulcerative gastritis and gastric obstruction that could result in impaired digestion, malnutrition, and increased susceptibility to comorbidities, among others. Furthermore, it has been described that juvenile pygmy sperm whales are susceptible to ingesting plastic debris, as deep-diving habits and early age are risk factors for this to occur, due to the fact that these animals have limited foraging experience and reduced ability to acoustically discriminate anthropogenic debris from prey [11]. According to ATR-FTIR analysis, all the plastic found in the stomach of the studied individual was low-density polyethylene, a polymer mainly used for packaging applications. The density of the polymer is below water density; therefore, once the plastic is released or lost into the marine environment, it will float until it degrades into smaller parts and/or biofouling attaches onto it surface, which could indicate that the ingestion happened in the water column, as previous works state [49].

Although there was no gross evidence of neurological alterations, it cannot be totally discarded that the initial stages of a neurological pathology could be responsible for the aberrant feeding behaviour, although according to the literature, the authors believe that there are two more probable alternative hypotheses that can explain the large quantities of plant and plastic debris in the stomach of the studied animal: (1) Accidental ingestion of detached floating seagrass leaves and macroalgal thalli drifting entangled with plastic debris, or (2) intentional ingestion of plant material within seagrass meadows and floating plastic items due to altered feeding behaviour. Although the first hypothesis is plausible, the reason for the animal to ingest the large quantity of plant and plastic debris needed to develop the described pathology is not explained. However, the second hypothesis would explain the severe stomach impaction suffered by the stranded juvenile *K. breviceps*. Although in the case reported herein these are only behavioural hypotheses due to the lack of evidence, previous works have described parallel behaviours in other odontecete species that could be applicable to the current study. For example, Krzyszczyk et al. (2013) [5] concluded that maternal loss by separation or death was the cause that lead juvenile dolphins unable to hunt on regular prey to ingest great quantities of plant material to feel sated, which consequently lead to stomach impaction and death of the animals, this being concordant with the characteristics of the case described herein. Interestingly, Kastelein & Lavaleije (1992) [15] witnessed a harbour porpoise regurgitate a combination of vegetal, organic and plastic debris similar to the stomach contents described in the present work and hypothesised that the ingestion of plant material could have been intentional, having the objective to induce vomiting and expel the plastic irritating material, as terrestrial carnivores do. Although there is not enough evidence or bibliographical references to conclude that the *K. breviceps* individual studied in the present work ingested the plant material in order to regurgitate the plastic debris already lodged in its stomach, a behavioural response should at least be considered. Nevertheless, the present work is the first report, as far as the authors are aware, of a *K. breviceps* individual ingesting such quantities of plant material. Although the ingestion of vegetation in Kogiids is highly improbable because the usual hunting depths of these species are devoid of vegetal life, this could indicate an already deteriorated health status for when the plant material was ingested. Furthermore, as discussed above, the presence of *P. henslowii* in the stomach content is concordant with this aberrant phenomenon.

## 5. Conclusions

This case demonstrates that severe gastric obstruction associated with ingestion of plastics and vegetation was the most probable cause of death. Although the behavioural mechanisms leading to ingestion remain uncertain, reduced foraging efficiency in a juvenile individual represents one plausible explanation. Further case reports are required to determine whether vegetation ingestion is an exceptional event or an underreported phenomenon in Kogiids.

## Figures and Tables

**Table 1:** Detailed information of items found in the keratinized stomach of the stranded *K. breviceps*.

Taxonomic Group	Species	Weight (g)	Relative Weight of Stomach Contents (%)
**Food Items**		50.31	39.74
Unidentified remains		45.34	
**Decapoda**			
Portunidae	*Polybius henslowii*	4.03	
Pasiphaediae	*Pasiphaea multidentata*	0.94	
**Non-food Items**		76.30	60.26
**Plant material**		31.10	24.56
Zosteraceae	*Zostera marina*	23.02	
Fucaceae	*Ascophyllum nodosum*	4.23	
Unidentified		3.85	
**Plastic items**		45.20	35.70

Bold text indicates the main categories in which the items found in the keratinized stomach of the *K. breviceps* were classified.

**Table 2:** Detailed information of the plastic fragments analysed. LDPE: low-density polyethylene, LLDPE: linear low-density polyethylene, PE: polyethylene.

Sample	Shape	Size (cm)	Polymer Type	Origin
1	Film	11 × 7	LDPE/LLDPE	Packaging
2	Film	31 × 9	LDPE/LLDPE	Packaging
3	Film	16 × 4	LDPE/LLDPE	Food Packaging
4	Film	46 × 17	LDPE/LLDPE	Packaging
5	Film	20 × 5	LDPE/LLDPE	Packaging
6	Film	41 × 9	LDPE/LLDPE	Packaging
7	Film	16 × 5	LDPE/LLDPE	Packaging
8	Film	17 × 6	LDPE/LLDPE	Packaging
9	Film	9 × 5	PE (acrylic traces)	Packaging
10	Film	22 × 1	LDPE/LLDPE	Packaging
11	Film	17 × 14	LDPE/LLDPE	Food packaging
12	Film	31 × 27	LDPE/LLDPE	Food packaging
13	Rope	60 × 0.2	LDPE/LLDPE	Packaging
14	Rope	101 × 0.3	LDPE/LLDPE	Packaging
15	Fibres	37 × 0.1	LDPE/LLDPE	Fishing
16	Rope	23 × 0.1	LDPE/LLDPE	Fishing

## Data Availability

Data will be made available under request.

## Supplementary Materials

The following supporting information can be downloaded at: https://www.mdpi.com/article/10.3390/ani16142194/s1, Table S1: Library match score of the ATR-FTIR measurements. Match index is measured as hit quality index (HQI); Figure S1: General view of the three main stages of the necropsy: (A) External analysis; (B) subcutaneous analysis and (C) analysis of the internal organs. Scale bar: the total length of the scale is 40 cm; Figure S2: Attenuated total reflectance spectrums of the second (A), third (B) and fifteenth (C) plastic samples. “O-H water” and “salt” labelled rectangles indicate the effect of seawater degradation, clearly observed in the fifteenth sample; Figure S3: Unidentified plant material recovered from the keratinized stomach of the pygmy sperm whale stranded in Basque Coast. (A) General view of all the material; (B) detailed view of the tip of the plant; and (C) detailed view of the base of the plant. Scale bars: Each white or black rectangle measures 1 cm in length; Figure S4: Attenuated total reflectance spectrum of the first plastic sample and the analysis performed to detect the main picks.

## Abbreviations

The following abbreviations are used in this manuscript:

ATR-FTIR	Attenuated total reflectance Fourier-transformed infrared
LDPE	Low-density polyethylene
LLDPE	Linear low-density polyethylene
