# Supplementary material for: First Documented Fatal Gastric Obstruction Associated with Ingestion of Plastics and Vegetation in a Juvenile Kogia breviceps
Source: Animals (Basel). 2026 Jul 15;16(14):2194. doi: 10.3390/ani16142194 (PMC13405992; doi:10.3390/ani16142194)
Supplement: Supplementary file 1 [file animals-16-02194-s001.zip › animals-4395191-supplementary.pdf]

Supplementary Table S1: Library match score of the ATR-FTIR measurements. Match index is measured as hit quality index (HQI)

Sample	HQI (%)	Comments
1	82	-
2	80	Spectrum shown (Supl. Fig. 2A)
3	85	Spectrum shown (Supl. Fig. 2B)
4	82	-
5	81	-
6	85	-
7	83	-
8	81	-
9	70	-
10	83	-
11	83	-
12	81	-
13	76	-
14	78	-
15	73	Spectrum shown (Supl. Fig. 2C)
16	80	

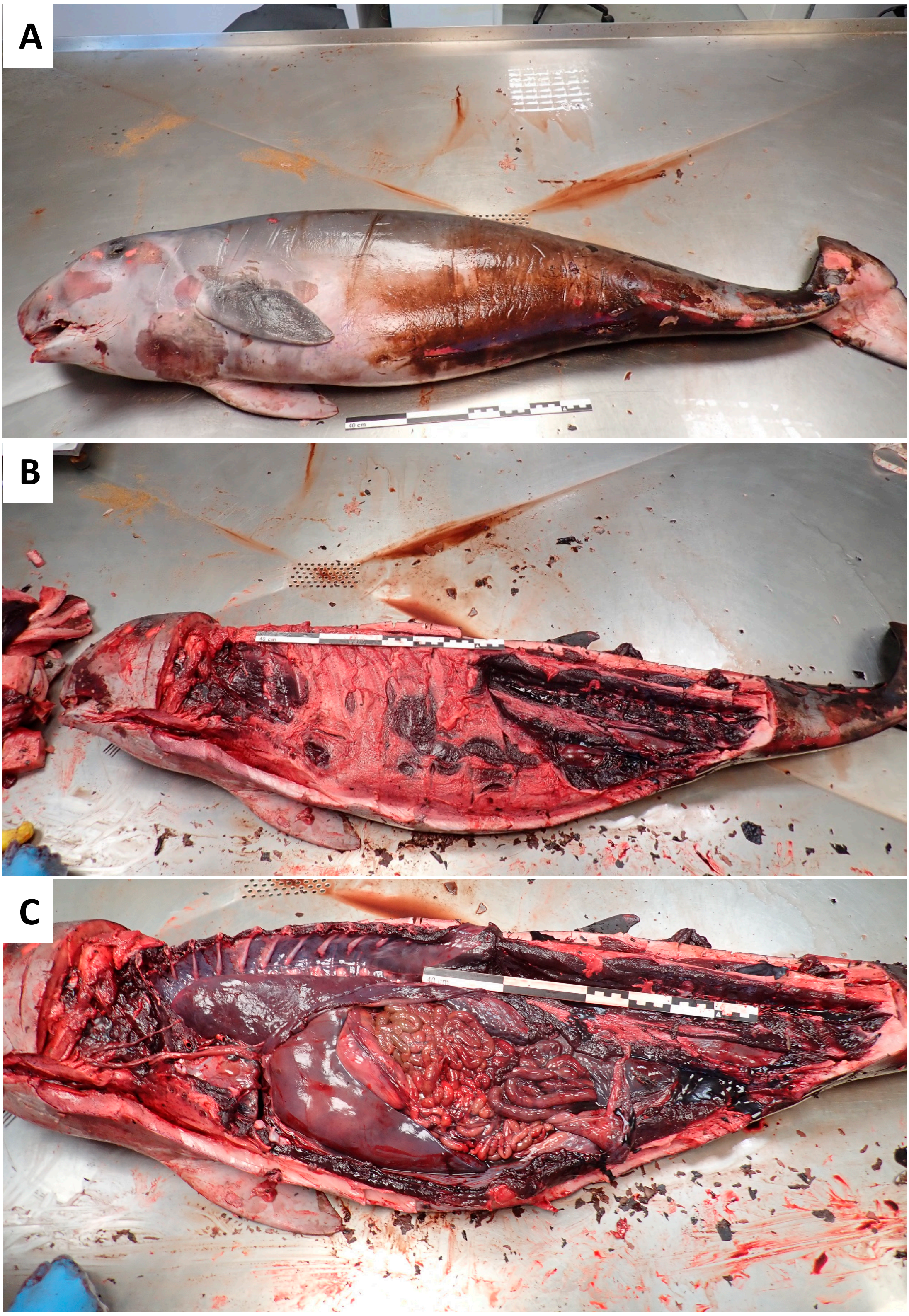

Supplementary Figure S1: General view of the three main stages of the necropsy: A) External analysis; B) subcutaneous analysis and C) analysis of the internal organs. Scale bar: the total length of the scale is 40 cm.

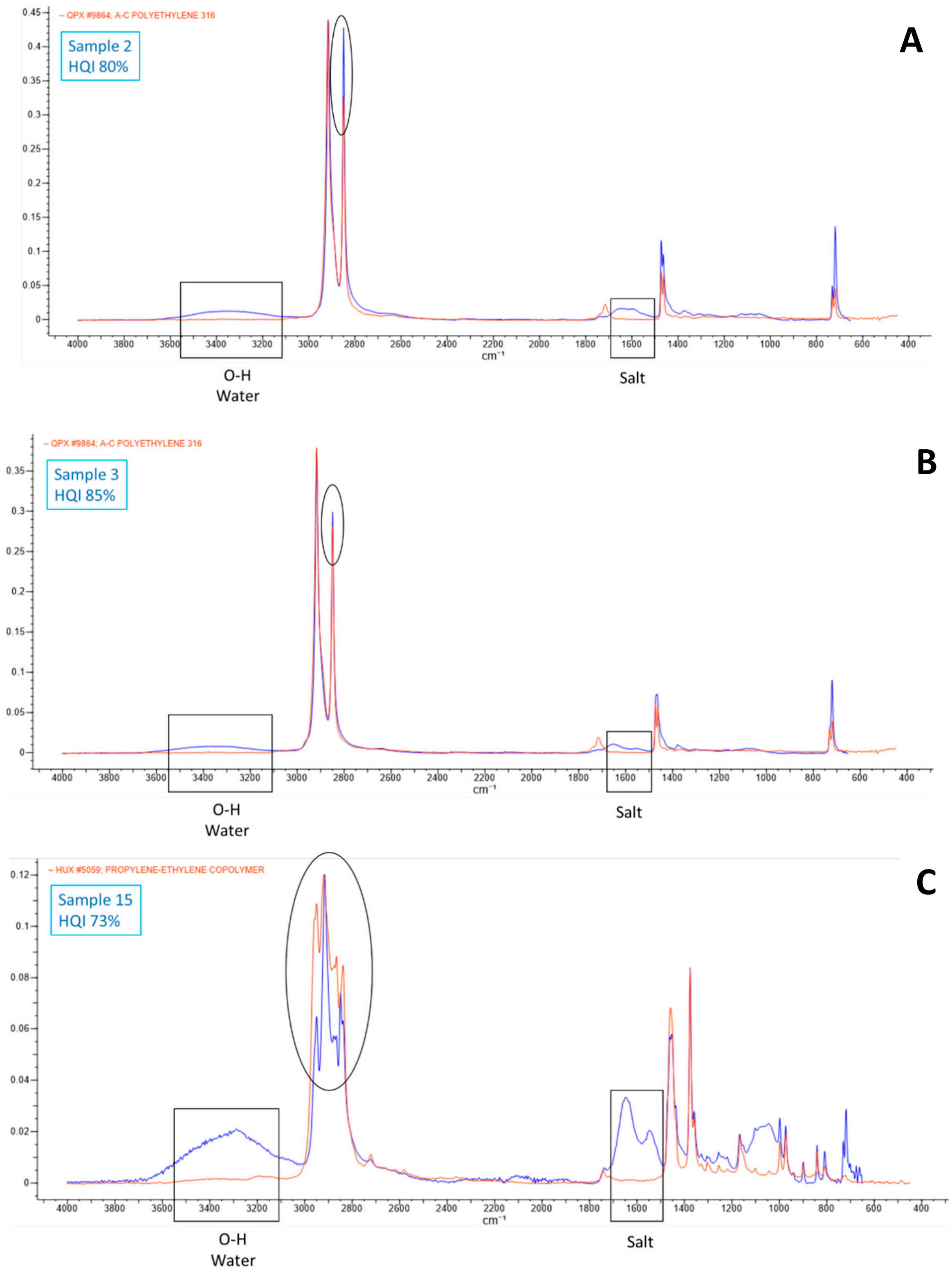

Supplementary Figure S2: Attenuated total reflectance spectrums of the second (A), third (B) and fifteenth (C) plastic samples. "O-H water" and "salt" labelled rectangles indicate the effect of seawater degradation, clearly observed in the fifteenth sample.

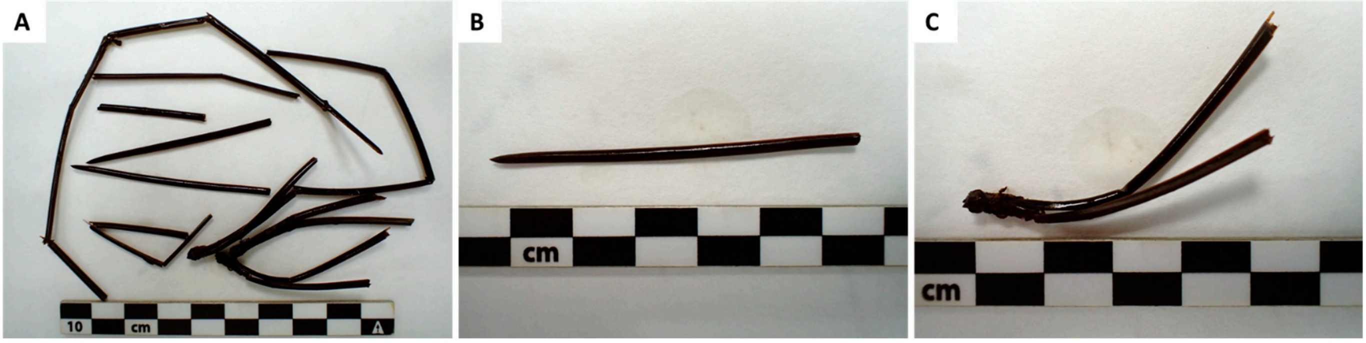

Supplementary Figure S3: Unidentified plant material recovered from the keratinized stomach of the pygmy sperm whale stranded in Basque Coast. A) General view of all the material; B) detailed view of the tip of the plant; and C) detailed view of the base of the plant. Scale bars: Each white or black rectangle measures 1 cm in length.

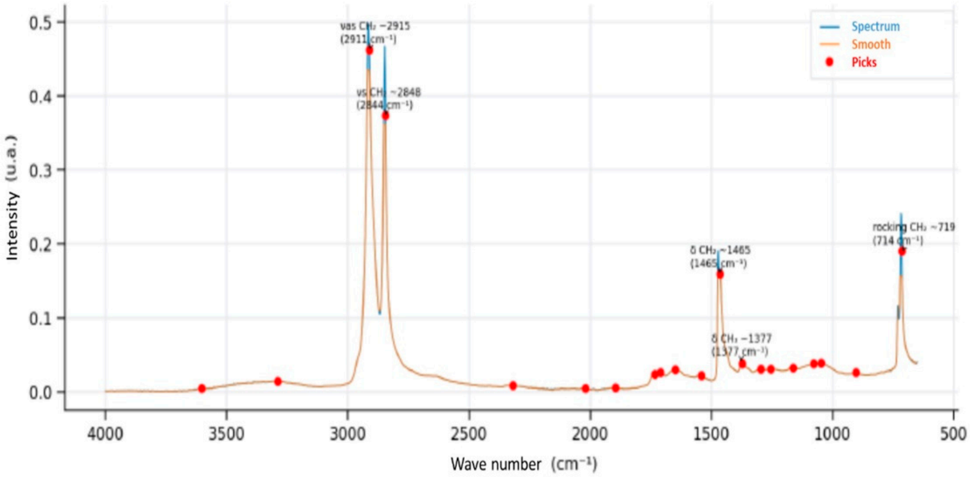

Supplementary Figure S4: Attenuated total reflectance spectrum of the first plastic sample and the analysis performed to detect the main picks.
